# Yeasts in sustainable bioethanol production: A review

**DOI:** 10.1016/j.bbrep.2017.03.003

**Published:** 2017-03-06

**Authors:** Siti Hajar Mohd Azhar, Rahmath Abdulla, Siti Azmah Jambo, Hartinie Marbawi, Jualang Azlan Gansau, Ainol Azifa Mohd Faik, Kenneth Francis Rodrigues

**Affiliations:** a Faculty of Science and Natural Resources, Universiti Malaysia Sabah, Jalan UMS, 88400 Kota Kinabalu, Sabah, Malaysia; b Energy Research Unit, Universiti Malaysia Sabah, Jalan UMS, 88400 Kota Kinabalu, Sabah, Malaysia; c Biotechnology Research Institute, Universiti Malaysia Sabah, Jalan UMS, 88400 Kota Kinabalu, Sabah, Malaysia

**Keywords:** Yeasts, Fermentation, Bioethanol, Immobilization

## Abstract

Bioethanol has been identified as the mostly used biofuel worldwide since it significantly contributes to the reduction of crude oil consumption and environmental pollution. It can be produced from various types of feedstocks such as sucrose, starch, lignocellulosic and algal biomass through fermentation process by microorganisms. Compared to other types of microoganisms, yeasts especially *Saccharomyces cerevisiae* is the common microbes employed in ethanol production due to its high ethanol productivity, high ethanol tolerance and ability of fermenting wide range of sugars. However, there are some challenges in yeast fermentation which inhibit ethanol production such as high temperature, high ethanol concentration and the ability to ferment pentose sugars. Various types of yeast strains have been used in fermentation for ethanol production including hybrid, recombinant and wild-type yeasts. Yeasts can directly ferment simple sugars into ethanol while other type of feedstocks must be converted to fermentable sugars before it can be fermented to ethanol. The common processes involves in ethanol production are pretreatment, hydrolysis and fermentation. Production of bioethanol during fermentation depends on several factors such as temperature, sugar concentration, pH, fermentation time, agitation rate, and inoculum size. The efficiency and productivity of ethanol can be enhanced by immobilizing the yeast cells. This review highlights the different types of yeast strains, fermentation process, factors affecting bioethanol production and immobilization of yeasts for better bioethanol production.

## Highlights

• Yeast strains applied in bioethanol production are highlighted.
• Fermentation in bioethanol production by yeasts are discussed.
• Factors that affect bioethanol production are considered.
• Immobilization of yeasts in bioethanol production are highlighted.

## 1 Introduction

The improvement of living standard urges the hunt for sustainable energy in order to meet energy consumption across the world [1]. On the other hand, the use of fossil fuels as the main energy resources caused the arising of worldwide problems such as environmental pollution and global warming [2], [3]. These led to the finding of environmentally friendly, renewable and sustainable energy by government, industrial and energy sector [4], [5]. Among renewable energies, priority was given to liquid biofuels as it represents about 40% of the total energy consumption in the world [6]. The use of liquid biofuels contributes to the reduction of greenhouse gas emissions, creation of job opportunities, regional development and supply security [5], [7].

Bioethanol is known as the most widely used biofuel in transportation sector and have a long history as alternative fuels. In 1984, Germany and France started to use bioethanol as a fuel in internal combustion engines (ICEs) [8]. Utilization of bioethanol by Brazil was initiated since 1925. In Europe and United States, bioethanol was widely used until the early 1900s. After World War II, the use of bioethanol was neglected due to its expensive production cost compared to petroleum fuel until the oil crisis in the 1970s [5]. The interest in using bioethanol has been increasing since the 1980s and it has been considered as an alternative fuel in many countries. Global ethanol production increased from 13.12 billions of gallons in 2007 to 25.68 billions of gallons in 2015 with a slight decreased in 2012 and 2013 [9]. United States is the largest ethanol producer with the production of nearly 15 billion gallons in 2015. The production of ethanol by United States and Brazil contribute to 85% world's ethanol production.

Bioethanol is also known as ethyl alcohol or chemically C_2_H_5_OH or EtOH. It can be used directly as pure ethanol or blended with gasoline to produce “gasohol” [10]. It can be used as a gasoline improver or octane enhancer and in bioethanol-diesel blends to reduce the emission of exhaust gasses [11]. Bioethanol offers several advantages over gasoline such as higher octane number (108), broader flammability limits, higher flame speeds and increased heats of vaporization [12]. In contrast to petroleum fuel, bioethanol is less toxic, readily biodegradable and produces lesser air-borne pollutants [13]. A variety of feedstocks from the first, second and third generation has been used in bioethanol production. The first-generation bioethanol involves feedstocks rich in sucrose (sugar cane, sugar beet, sweet sorghum and fruits) and starch (corn, wheat, rice, potato, cassava, sweet potato and barley). Second-generation bioethanol comes from lignocellulosic biomass such as wood, straw and grasses. Third-generation bioethanol has been derived from algal biomass including microalgae and macroalgae [14].

Microorganisms such as yeasts play an essential role in bioethanol production by fermenting a wide range of sugars to ethanol. They are used in industrial plants due to valuable properties in ethanol yield (>90.0% theoretical yield), ethanol tolerance (>40.0 g/L), ethanol productivity (>1.0 g/L/h), growth in simple, inexpensive media and undiluted fermentation broth with resistance to inhibitors and retard contaminants from growth condition [15]. As the main component in fermentation, yeasts affect the amount of ethanol yield. In this review, the role of yeasts in bioethanol fermentation and its immobilization techniques will be discussed in order to enhance the production of ethanol for the benefits of mankind.

## 2 Yeasts

Yeasts are defined as ascomycetous or basidiomycetous fungi that are capable of reproducing by budding or fission and form spores which are not enclosed in a fruiting body [16]. They are first classified based on its sexuality (Ascomycotina or Basidiomycotina) or the lack of sexual phase in the life cycle (Deuteromycotina). The lower taxonomic subdivisions (families, subfamilies, genera, species and strain) are determined by its morphological, physiological and genetic characteristics including sexual reproduction [17].

### 2.1 Yeasts diversity

The number of discovered yeasts has been increasing from year to year. More than 2500 yeast species were published by 2005. It is assumed that only 1% of yeast species is currently known which represents approximately 1500 species. The total numbers of yeast species on earth are expected to reach 150,000 [18]. The diversity of yeast species in particular niches is determined by its capability of utilizing different carbon source and its nutritional selectivity as it exhibits great specialization for habitat [19]. Yeasts can be isolated from the terrestrial, aquatic and aerial environment. Plant is the preferred habitat of yeasts community. A few species are found to have commensalism or parasitic relationships with animals. Extreme environments like low water potential (high sugar or salt concentration) and low temperature may be inhabited by yeasts [20], [21]. The natural habitats of yeasts are summarized in Table 1.

**Table 1:** Natural yeasts habitats [20], [21].

**Habitat**	**Description**	**Yeasts genera**
Plants	• The common niche of yeasts is the interface between soluble nutrients of plants (sugars) and the septic world • insects help in spreading yeasts on the phyllosphere	*Ashbya* spp.
		*Nematospora* spp.
Animals	• Several yeasts are pathogenic toward humans and animals while others are non-pathogenic (can be found in intestinal tract and skin of warm-blooded animals) • Numerous yeasts are commensal to insect which act as vectors for natural distribution of yeasts.	*Candida* spp.
		*Cyniclomyces* spp.
		*Pityrosporum* spp.
Soil	• Considered as reservoir for yeasts long-term survival rather than habitat for free growth • Yeasts can be found only in the aerobic soil layers (10–15 cm)	*Lipomyces* spp.
		*Schwanniomyces* spp.
Water	• Yeasts can be found in both fresh water and seawater • Estuarine regions usually have higher numbers of yeasts compared to seawater	*Rhodotorula* spp.
		*Debaryomyces* spp.
Atmosphere	• Yeasts are dispersed by air currents from the vegetative layer above soil surfaces • Only a few yeasts may be expected per volume of air	*Cryptococcus* spp.
		*Rhodotorula* spp.
		*Sporobolomyces* spp.
		*Debaryomyces* spp.
Extreme environment	• Some halotolerant yeasts can grow in nearly saturated brine solution • Osmophilic yeasts were discovered in glacier horizons	*Debaryomyces* spp.
		*Zygosaccharomyces* spp.

There are a broad diversity of yeast cells including its size, shape and colour. Cell sizes of yeasts are influenced by its species and growth condition. The length of some yeast cells are only 2–3 µm while the other species may reach the length of 20–50 µm [19]. Most yeasts have a width in the range of 1–10 µm. Generally, the sizes of brewing strains of *S. cerevisiae* are larger than laboratory strains [22]. Many yeast species including *Saccharomyces* spp. are ellipsoidal or ovoid in shape and have creamy colour colonies [20], [21].

### 2.2 Molecular genetics of yeasts

The production of bioethanol is founded on the ability of yeasts to catabolize six-carbon molecules such as glucose into two carbon components, such as ethanol, without proceeding to the final oxidation product which is CO_2_. Crabtree positive yeasts such as *S. cerevisiae* accumulate ethanol in the presence of oxygen, however *Candia albicans* which is a crabtree-negative yeast catabolizes sugars into CO_2_ in the presence of oxygen [23]. The presence of six carbon carbohydrates represses the oxidative respiration pathway in Crabtree positive yeasts and energy for growth is generated via glycolysis. Upon depletion of six carbon molecules, the catabolism shifts to oxidation of two carbon molecules into CO2 [24]. This phenomenon is termed at the ‘diauxic shift’. The process of bioethanol production via fermentative metabolism and the diauxic shift is dependent upon the enzyme Alcohol Dehydrogenase (EC 1.1.1.1) which is encoded on the *ADH1*locus. ADH1 catalyzes the reduction of acetaldehyde to ethanol during the fermentation of glucose, it can also catalyze the reverse reaction which is the conversion of ethanol into acetaldehyde, albeit with a lower catalystic efficiency [25].

The yeast S. *cerevisiae* contain two genes that encode ADH, *ADH1* is expressed constitutively, while the expression of *ADH2* is induced by the reduction in the intracellular concentration of glucose. The substrate for the enzyme ADH2 is ethanol [26]. The expression of ADH2 gene is governed by transcription factors and genome sequencing and transcriptome analysis has revealed the structure and DNA binding elements of these regulatory proteins [27]. Recent advances in synthetic biology have focused on re-engineering the ADH gene for greater substrate specificity and improvement of catalytic activity as well as engineering the yeast genome with protein coding genes [28] which improve tolerance to ethanol and catalysis of a wide range of carbon sources [29]. Molecular biologists are actively seeking novel genes encoding ADHs using metagenomic approaches, and this had yielded a number of unique variants [30].

### 2.3 Yeasts in bioethanol production

Since thousands of years ago, yeasts such as *S. cerevisiae* have been used in alcohol production especially in the brewery and wine industries. It keeps the distillation cost low as it gives a high ethanol yield, a high productivity and can withstand high ethanol concentration [31]. Nowadays, yeasts are used to generate fuel ethanol from renewable energy sources [32]. Certain yeast strains such as *Pichia stipitis* (NRRL-Y-7124), *S. cerevisiae* (RL-11) and *Kluyveromyces fagilis* (Kf1) were reported as good ethanol producers from different types of sugars [33].

*S. cerevisiae* is the most commonly employed yeast in industrial ethanol production as it tolerates a wide range of pH [34] thus making the process less susceptible to infection. Baker's yeast was traditionally used as a starter culture in ethanol production due to its low cost and easy availability. However, baker's yeast and other *S. cerevisiae* strains were unable to compete with wild-type yeast which caused contamination during industrial processes. Stressful conditions like an increase in ethanol concentration, temperature, osmotic stress and bacterial contamination are the reasons why the yeast cannot survive during the fermentation [35]. Flocculent yeasts were also used during biological fermentation for ethanol production as it facilitates downstream processing, allows operation at high cell density and gives higher overall productivity [36], [37]. It reduces the cost of cells recovery as it separate easily from the fermentation medium without centrifugation [38].

There are common challenges to yeasts during sugar fermentation which are rise in temperature (35–45 °C) and ethanol concentration (over 20%) [39]. Yeasts growth rate and metabolism increase as the temperature increases until it reaches the optimum value. Increase in ethanol concentration during fermentation can cause inhibition to microorganism growth and viability [40], [41]. Inability of *S. cerevisiae* to grow in media containing high level of alcohol leads to the inhibition of ethanol production [42]. The other problems in bioethanol fermentation by yeast are the ability to ferment pentose sugars. *S. cerevisiae* is the most commonly used in bioethanol production. However, it can only ferment hexoses but not pentoses [43]. Only some yeasts from genera *Pichia*, *Candida*, *Schizosaccharomyces* and *Pachysolen* are capable of fermenting pentoses to ethanol [33].

The efficiency of ethanol production on an industrial scale will be increased by using yeasts that are tolerant to inhibitors [39]. The common challenges of yeasts can be overcome by using ethanol-tolerant and thermotolerant yeast. Ethanol-tolerant and thermotolerant strains which can resist stresses can be isolated from natural resources such as soil, water, plants and animals. This is because cells adapt to their environment over time by natural selection. Ethanol fermentation at high temperature is a beneficial process as it selects thermo-tolerant microorganisms and does not require cooling costs and cellulase [44]. For example, *K. Marxianus* is thermotolerant yeast which is capable of co-fermenting both hexose and pentose sugars and can survive the temperature of 42–45 °C [45].

The problems of pentose fermentation can be solved by using hybrid, genetically engineered or co-culture of two yeast strains. Hybrid yeast strains are used simultaneously to ferment pentose and hexose sugars to ethanol. The hybrid strain has been developed by fusing protoplast of *S. cerevisiae* and xylose-fermenting yeasts like *P. tannophilus*, *C. shehatae* and *P. stipitis* [46]. Genetically engineered *S. cerevisiae* and co-culture of two strains have been developed to produce bioethanol from xylose with high yield. Genetic engineering use recombinant DNA technology to up-regulate the stress tolerance genes in order to overcome the inhibitory situations [47]. Xylose reductase and xylitol dehydrogenase genes from *S. stipitis* were introduced into *S. cerevisiae* to develop strain with the ability of fermenting xylose. The engineered yeast strains can convert cellulose to ethanol more rapidly compared to unmodified yeast strains. Co-culture process simultaneously culture and grow two different yeasts in the same reactor [48]. Co-culture shows better ethanol production as compared to its pure culture [49]. In co-culture, pentose utilizing yeasts like *Pichia fermentans* and *Pichia stipitis* are combined together with *S. cerevisiae* so that hexose and pentose sugars can be efficiently utilized [50], [51].

Yeast strains that have been used in bioethanol production are summarized in Table 2. *S. cerevisiae* was the most widely studied yeasts. Different types of feedstock were used for the production of bioethanol. Kim et al. [52] reported the highest ethanol concentration of 96.9g/L with a productivity of 3.46g/L/h. It was contributed by the wild-type yeast strain used, *S. cerevisiae* KL17 which is capable of utilizing both glucose and galactose simultaneously. It shows that wild-type yeasts has high potential in fermenting sugars to ethanol. Moreover, Silva-filho et al., [53] reported that wild-type strains could be more efficient to the industrial process than commercial strains. Fermentation of giant reed using *S. stipitis* CBS 6054 obtained the lowest ethanol concentration of 8.2g/L with a productivity of 0.17g/L/h [54]. At the optimum condition for sugars release, the levels of toxic degradation products exceed the critical level and made the condition unsuitable for yeast fermentation.

**Table 2:** Yeast strains used in bioethanol production.

**Yeast strain**	**Type of strain**	**Feedstock**	**Sugar concentration (g/L)**	**Fermentation condition**	**Ethanol concentration (g/L)**	**Ethanol productivity (g/L/h)**	**References**
*S. cerevisiae* RL-11	Laboratory	Spent coffee grounds	195.0	30 °C, 200 rpm, 48 h	11.7	0.49	[33]
*S. cerevisiae* MTCC 173	Laboratory	Sorghum stover	200.0	30 °C, 120 rpm, 96 h	68.0	0.94	[109]
*S. stipitis* CBS 6054	Laboratory	Giant reed	33.4	30 °C, 150 rpm, 96 h	8.2	0.17	[54]
*S. cerevisiae* KL17	Wild-type	Galactose and glucose	500.0	30 °C, 200 rpm, 28 h	96.9	3.46	[52]
*S. pombe* CHFY0201	Wild-type	Cassava starch	95.0	32 °C, 120 rpm, 66 h	72.1	1.16	[110]
*S. cerevisiae* CHY1011	Wild-type	Cassava starch	195.0	32 °C, 120 rpm, 66 h	89.1	1.35	[111]
*S. cerevisiae* ZU-10	Recombinant	Corn stover	99.0	30 °C, 180 rpm, 72 h	41.2	0.57	[112]
*S. cerevisiae* RPRT90	Mutated hybrid	*Ipomea carnea*	72.1	30 °C, 150 rpm, 28 h	29.0	1.03	[46]
*S. cerevisiae* CHFY0321(protoplast fusant)	Hybrid	Cassava starch	195.0	32 °C, 120 rpm, 65 h	89.8	1.38	[38]

## 3 Process in bioethanol production

The process of ethanol production depends on the types of feedstocks used. Generally, there are three major steps in ethanol production: (1) obtaining solution that contains fermentable sugars, (2) converting sugars to ethanol by fermentation and (3) ethanol separation and purification [108]. Feedstocks are usually pretreated in order to reduce its size and facilitate subsequent processes. Then, the hemicellulose and cellulose will be hydrolyzed to fermentable sugars. Yeasts are given the responsibility to ferment these sugars into ethanol. Separation technologies are used to recover ethanol before it can be used as fuel [55].

### 3.1 Pretreatment

Pretreatment has a significant effect on the overall process which makes the hydrolysis easier and produces higher amount of fermentable sugars. It influences the amount of ethanol yield and production cost [56]. Methods that are currently used for pretreatments are physical, chemical, biological and physicochemical. Physical pretreatment uses mechanical milling to ground the substrate. The common chemical pretreatment includes ozonolysis, acid hydrolysis, alkaline hydrolysis [57] and organosoly based process [58]. Different fungal species are involved in biological pretreatment while physicochemical pretreatment includes ammonia fibre explosion [59] and steam [60]. Dehydration of hexose and pentoses during pretreatment release furan compounds like 5-hydroxymethyl-2-furaldehyde (HMF) and 2-furaldehyde. These furan derivatives induce the inhibition of cell growth and reduce ethanol productivity [61]. Yeasts fermentation is inhibited by the weak acid stress induced from lignocellulosic materials. However, the low concentration of weak acids can increase ethanol production by cellular division. It was reported that the presence of weak acids can improve glucose utilization, ethanol production and tolerance to HMF and furfural in *S. cerevisiae* [62].

### 3.2 Hydrolysis

Hydrolysis process takes place after pretreatment to break down the feedstocks into fermentable sugar for bioethanol production. The two most commonly used hydrolysis methods are acidic and enzymatic. Acid hydrolysis is considered as the oldest and most commonly used method [63]. Acidic hydrolysis can be divided into two types namely dilute and concentrated. Dilute acid hydrolysis is performed at higher temperature using low acid concentration while concentrated acid hydrolysis is carried out at lower temperature using high acid concentration. Dilute acid hydrolysis is the most commonly used process. However, it generates large amount of inhibitors compared to concentrated acid hydrolysis. Acid hydrolysis of lignocellulosic biomass is conducted in two-stage process as the pentose sugars degrade more rapidly compared to hexose sugars. Hemicellulose is hydrolyzed in the first stage using dilute acid while cellulose is hydrolyzed in the second stage using concentrated acid. Concentrated acid process generates high sugar recovery (90%) in shorter period of time [64]. The disadvantages of acid hydrolysis are the difficulty of performing acid recovery and recycling process which increases the production cost.

Enzymatic hydrolysis requires enzymes to hydrolyse the feedstocks into fermentable sugars. Three types of enzymes that are commonly used for cellulose breakdown such as endo-β—1,4-glucanases, cellobiohydrolases and β-glucosidases. The activity of cellulase enzyme is influenced by the concentration and source of the enzyme. Cellulose will be degraded into reducing sugars under mild reaction conditions (pH: 4.8–5.0, temperature: 45–50 °C). Moreover, it does not cause corrosion problem in the reactors which can result in high sugar yields. The efficiency of enzymatic hydrolysis is influenced by optimized conditions such temperature, time, pH, enzyme loading and substrate concentration [65]. The amount of fermentable sugar obtained increases as the enzyme load increases while cellulose load decreases. Enzymatic saccharification of cellulose can be enhanced by using surfactants which function to block lignin. The efficiency of cellulose hydrolysis can be improved by adding Polyethylene glycol (PEG) or Tween 20 to increase enzymatic saccharification and reduce the adsorption of cellulase on lignin [64]. The limitation of using enzymes in hydrolysis is because they are too expensive for the economical production of ethanol from biomass.

### 3.3 Fermentation process

There are three processes that are commonly used in bioethanol production which are separate hydrolysis and fermentation (SHF), simultaneous saccharification and fermentation (SSF) and simultaneous saccharification and co-fermentation (SSCF). In SHF, hydrolysis of lignocellulosic materials is separated from ethanol fermentation. The separation of enzymatic hydrolysis and fermentation allows enzyme to be operated at high temperature for better performance while fermentation organisms can be operated at moderate temperature for optimizing sugar utilization. SSF and SSCF have a short overall process as the enzymatic hydrolysis and fermentation process occur simultaneously to keep the concentration of glucose low. For SSF, the fermentation of glucose is separated from pentoses while SSCF ferment glucose and pentoses in the same reactor [65]. Both SSF and SSCF are preferred over SHF because the operation can be performed in the same tank. The benefits of both processes are lower cost, higher ethanol yield and shorter processing time [66].

Fermentation of bioethanol can be carried out in batch, fed-batch, repeated batch or continuous mode. In batch process, substrate is provided at the beginning of the process without addition or removal of the medium [67]. It is known as the simplest system of bioreactor with multi-vessel, flexible and easy control process. The fermentation process is carried out in a closed-loop system with high sugars and inhibitors concentration at the beginning and ends with high product concentration [68]. There are several benefits of batch system including complete sterilization, does not require labour skills, easy to manage the feedstocks, can be can be control easily and flexible to various product specifications [69], [70]. However, the productivity is low and need intensive and high labour costs. The presence of high sugar concentration in the fermentation medium may lead to substrate inhibition and results in the inhibition of cell growth and ethanol production [71].

Cell recycle batch fermentation (CRBF) is a strategic method for effective ethanol production as it reduce time and cost for inoculum preparation. The other advantages of repeated-batch process are easy cell collection, stable operation and long-term productivity [72], [73]. Sugar materials and immobilized yeast cells are used to facilitate cell separation for cell recycling [74], [75]. Combination of SSF and repeated-batch fermentation has been successfully applied on the fermentation of cassava starch using flocculating yeast [76]. However, its application in SSF process of lignocellulosic materials is extremely difficult because lignocelluosic residue remain in the fermentation medium together with yeast cells [77]. The use of free cells in this system reduces yeast cell concentration and results in lower ethanol production in the subsequent batches. Repeated-batch fermentation can be performed by replacing free cells with the immobilized cells [78].

Fed-batch fermentation is a combination of batch and continuous mode which involves the addition of substrate into the fermentor without removing the medium. It has been used to overcome the problem of substrate inhibition in batch operation. Volume of culture in fed-batch processes can vary widely but it must be fed properly at certain rate with the right component composition. Productivity of fed-batch fermentation can be increased by maintaining substrate at low concentration which allows the conversion of sufficient amount of fermentable sugars to ethanol [70]. This process has higher productivity, higher dissolved oxygen in medium, shorter fermentation time and lower toxic effect of the medium components compared to other types of fermentation [71]. However, ethanol productivity in fed-batch is limited by feed rate and cell mass concentration [79]. Fed-batch operation has been applied successfully in non-uniform SSF system by continuously adding a pretreated substrate in order to achieve relatively high sugar and ethanol concentration [80].

Continuous operation is carried out by constantly adding substrates, culture medium and nutrients into a bioreactor containing active microorganisms. Culture volume in continuous operation must be constant and the fermentation products are taken continuously from the media. Various type of products can be obtained from the top of the bioreactor such as ethanol, cells and residual sugar [69]. The advantages of continuous system over batch and fed-batch system are higher productivity, smaller bioreactor volumes and less investment and operational costs [70]. At high dilution rate, ethanol productivity is increased while ethanol yield is decreased due to incompletely substrate consumption by yeasts [81]. However, the possibility for contamination to occur is higher than other types of fermentation [66]. Moreover, the ability of yeasts to produce ethanol in continuous process are reduced due to long cultivation time.

Processes involved in bioethanol production are summarized in Table 3. Enzymatic hydrolysis is the preferred saccharification method because of its higher yields, higher selectivity, lower energy cost and milder operating condition than chemical processes [82]. The most commonly used pretreatment method is steam explosion. This is contributed by the attractive features of steam explosion which has less environmental impact, low capital investment, high energy efficiency, less hazardous process chemicals and conditions and complete sugar recovery [83]. Fermentation of *Miscanthus* by *S. cerevisiae* CHY1011 reported the highest ethanol concentration of 69.2g/L with a productivity of 1.24g/L/h [84]. The pretreatment method applies a high shearing force to increase the biomass surface and results in increased enzymatic digestibility. Moreover, the continuous SSF feeding system increased the biomass concentration thus providing sufficient time for liquefaction of the substrate by the enzyme. Scordia et al. [85] reported the lowest ethanol concentration of 12.1g/L with a productivity of 0.13g/L/h by fermenting *Miscanthus x giganteus* using *S. stipitis* CBS 6054. This is because sugar recovery from the water soluble fraction (WSF) which has been used as the feedstock for fermentation was low.

**Table 3:** Processes involved in bioethanol production.

**Yeast strain**	**Feedstock**	**Pretreatment**	**Fermentation condition**	**Ethanol concentration (g/L)**	**Ethanol Productivity (g/L/h)**	**References**
*S. cerevisiae* CHY1011	*Miscanthus sacchariflorus*	CHEMET with sodium hydroxide	Continuous SSF, 33 °C, 56 h, 25% WIS	69.2	1.24	[84]
*S. cerevisiae* TMB3400	Wood chips	Steam explosion	Batch SSCF, 3 °C, 96 h, 8% WIS	32.9	0.34	[113]
*S. cerevisiae* ATCC24858	Reed	Phosphoric acid-acetone	Batch SSF, 38 °C, 150 rpm, 96 h, 36.1% WIS	55.5	0.57	[114]
*S. cerevisiae* VIT C-10880	*Arundo donax*	Steam explosion	Enzymatic hydrolysis, 45 °C, 72 h; Batch SHF, 32 °C, 500 rpm, 96 h, 10% WIS	20.6	0.21	[115]
*S. cerevisiae*	Reed	Liquid hot water	Enzymatic hydrolysis, 50 °C, 18 h; Semi fed-batch SSF, 36 °C, 60 h, 10% WIS	39.4	0.66	[116]
*S. cerevisiae* TMB 3400	Wheat meal and wheat straw	Steam explosion	Enzymatic hydrolysis, 40 °C, 850 rpm, 120 h; Fed-batch SHCF, 32 °C, 300 rpm, 120 h, 7.5% WIS	53.3	0.44	[117]
*S. cerevisiae*	*Liriodendron tulipifera*	Acid-free organosolv	Batch SSF, 30 °C, 150 rpm, 96 h, 1% WIS	29.9	0.42	[118]
Baker's yeast	Corn stover	Steam explosion	Fed-batch SSF, 30 °C, 700 rpm, 72 h, 10% WIS	25.7	0.36	[119]
*S. stipitis* CBS 6054	*Miscanthus giganteus*	Dilute oxalic acid	SSF, 30 °C, 96 h, 10% WSF	12.1	0.13	[85]
*S. cerevisiae*	Industrial hemp	Steam explosion	SSF, 37 °C, 348 rpm, 72 h, 7.5% WIS	21.3	0.30	[120]

### 3.4 Factors affecting bioethanol production

There are several factors which influence the production of bioethanol including temperature, sugar concentration, pH, fermentation time, agitation rate, and inoculum size [86]. The growth rate of the microorganisms is directly affected by the temperature [87]. High temperature which is unfavorable for cells growth becomes a stress factor for microorganisms [88]. The ideal temperature range for fermentation is between 20 and 35 °C. Free cells of *S. cerevisiae* have an optimum temperature near 30 °C whereas immobilized cells have slightly higher optimum temperature due to its ability to transfer heat from particle surface to inside the cells [89]. Moreover, enzymes which regulate microbial activity and fermentation process are sensitive to high temperature which can denature its tertiary structure and inactivates the enzymes [90]. Thus, temperature is carefully regulated throughout the fermentation process.

The increase in sugar concentration up to a certain level caused fermentation rate to increase. However, the use of excessive sugar concentration will cause steady fermentation rate. This is because the concentration of sugar use is beyond the uptake capacity of the microbial cells. Generally, the maximum rate of ethanol production is achieved when using sugars at the concentration of 150 g/L. The initial sugar concentration also has been considered as an important factor in ethanol production. High ethanol productivity and yield in batch fermentation can be obtained by using higher initial sugar concentration. However, it needs longer fermentation time and higher recovery cost [86].

Ethanol production is influenced by pH of the broth as it affects bacterial contamination, yeast growth, fermentation rate and by-product formation. The permeability of some essential nutrients into the cells is influenced by the concentration of H^+^ in the fermentation broth [86]. Moreover, the survival and growth of yeasts is influenced by the pH in the range of 2.75–4.25 [91]. In fermentation for ethanol production, the optimum pH range of *S. cerevisiae* is 4.0–5.0 [34]. When the pH was below than 4.0, a longer incubation period is required but the ethanol concentration was not reduced significantly. However, when then pH was above 5.0, the concentration of ethanol reduced substantially [10].

Fermentation time affect the growth of microorganisms. Shorter fermentation time causes inefficient fermentation due to inadequate growth of microorganisms. On the other hand, longer fermentation time gives toxic effect on microbial growth especially in batch mode due to the high concentration of ethanol in the fermented broth. Complete fermentation can be achieved at lower temperature by using longer fermentation time which results in lowest ethanol yield [86].

Agitation rate controls the permeability of nutrients from the fermentation broth to inside the cells and removal of ethanol from the cell to the fermentation broth. The greater the agitation rate, the higher the amount of ethanol produced. Besides, it increases the amount of sugar consumption and reduces the inhibition of ethanol on cells. The common agitation rate for fermentation by yeast cells is 150–200 rpm. Excess agitation rate is not suitable for smooth ethanol production as it causes limitation to the metabolic activities of the cells [86].

Inoculum concentration does not give significant effects on the final ethanol concentration but it affects the consumption rate of sugar and ethanol productivity [92]. The production of ethanol was seen to be increased with the increase in cell numbers from 1×10^4^ to 1×10^7^ cells per ml but there was no significant ethanol production found between 10^7^ and 10^8^ cells per ml. This is because the increase in cell concentration within certain range reduces fermentation time as the cells grow rapidly and directly consumes sugars into ethanol [86].

Factors affecting the production of bioethanol are shown in Table 4. Most of fermentation process using *S. cerevisiae* was carried out the at 30 °C whereas fermentation using *K. marxianus* was performed at 42 °C. The ideal temperature for bioethanol production depends on the ideal temperature of the yeasts. Most of the fermenting medium used for bioethanol production has pH in the range of 4.5–5.5 with various sugar concentration. Fermentation process is commonly performed at 24 and 72 h with rotation at 120 and 150 rpm. The common inoculum size employed in bioethanol production are 5% and 10%. Zhang et al. [93] reported the highest ethanol concentration (128.5g/L) and ethanol productivity (4.76g/L/h) probably due to favourable conditions for the yeast to produce bioethanol. The lowest ethanol concentration (9.5g/L) and ethanol productivity (0.31g/L/h) was produced from water hyacinth due to its low sugar concentration which limits substrate for bioethanol production [94].

**Table 4:** Factors affecting bioethanol production.

**Yeast strain**	**Feedstock**	**Temperature, (**°**C)**	**pH**	**Time (h)**	**Sugar concentration (g/L)**	**Agitation rate (rpm)**	**Inoculum size (%)**	**Ethanol concentration (g/L)**	**Ethanol Productivity (g/L/h)**	**References**
*Saccharomyces cerevisiae* CHY1011	Cassava starch	32	4.5	66	585.0	120	5	89.1	2.10	[111]
*Saccharomyces cerevisiae* ZU-10	Corn stover	30	5.5	72	99.0	120	5	41.2	0.57	[112]
*Saccharomyces cerevisiae* K35	Instant noodle waste	30	–	24	84.0	250	5	41.3	1.72	[121]
*Saccharomyces cerevisiae*	Wood	30	5.5	16	37.47	150	10	18.52	1.16	[122]
*Saccharomyces cerevisiae ATCC #24858*	Reed	38	5.0	96	123.0	150	10	55.0	0.57	[114]
*Saccharomyces cerevisiae*	Sweet potato	30	5.3	24	240.0	150	7	128.5	4.76	[93]
*Kluyveromyces marxianus K213*	Water hyacinth	42	4.8	24	23.3	–	5	7.34	0.31	[94]
*Kluyveromyces marxianus CECT 10875*	Wheat straw	42	5.5	72	–	150	–	36.2	0.50	[123]
*Saccharomyces cerevisiae* GIM-2	Paper sludge	33	–	16	27.8	60	6	9.5	0.59	[124]
*Saccharomyces cerevisiae* CHFY0321	Cassava mash	33	–	42	183.5	100	5	86.1	2.41	[125]

A large amount of ethanol must be produced in order to fulfill the increasing worldwide demand. However, the production of ethanol using free yeast cells is still inefficient due to its higher cost of cell cycling, greater contamination risk, limitation of the dilution rate and susceptibility to environmental variations [95]. Moreover, free cells cause substrate or product inhibition from direct contact between the cells and medium. Most of the problems occurred in free-cell systems are reduced by the immobilization method.

## 4 Immobilization

Immobilized cell technology is commonly applied in fermentation process. The benefits of immobilized cells over free cells include higher cell density per volume of reactor, easier separation from the reaction medium, higher substrate conversion, less inhibition by products, shorter reaction time and control of cell replication [96]. The immobilization of yeast cells and its productivity are influenced by several factors such as the surface characteristics of the carrier, pore size, water content, hydrophilicity and magnetism [97]. Immobilization should be performed under mild condition to maintain the activity of the cells [98].

### 4.1 Immobilization of yeast cells

Cells can be immobilized by different types of methods like adsorption, crosslinking, encapsulation and entrapment. Entrapment is carried out by the polymerization of an aqueous solution of acrylamide monomers in which microorganisms are suspended. It is commonly used to overcome the problems of degradation and limitation of mass transfer. It avoids the release of cells while allowing diffusion of substrates and products [99]. This method allows high biomass loading which results in high ethanol productivity. Entrapment method is widely used due to its simplicity, non-toxic, less expensive, reversible and good mechanical properties. Entrapment can be operated at extremely high dilution rates without causing washout of cells. Most of the researches involving the immobilization of microbial cells were focused on gel entrapment. The most commonly used gels are in the form of spherical beads with diameters in the range of 0.3–5 mm. However, gel has limited mechanical stability which can be easily damaged by the growth of the microbial cells and carbon dioxide production. Moreover, the presence of phosphates causes the weakening of calcium alginate gels [79].

Adsorption is a very popular way of cell immobilization due to its simple, cheap and fast method. Cells are attached to the surface of the material by electrostatic force such as Van der Waals forces, ionic bonds, hydrogen bridges or covalent interactions. Ionic attraction is used to immobilize yeast cells. The supporting material used must have a high affinity in order for the yeast strain to withstand the environmental conditions present within the bioreactor. In most cases of continuous ethanol production, adsorption is carried out by circulating a concentrated suspension of yeast cells through the bioreactor for several hours. Adsorption technique does not require the use of toxic chemical and the yeast cells can be maintained in a viable state. The absorbed-cell system is limited by lower biomass loading and lower feed flow rates compared to entrapped-cell system. This is because the number of yeast cells that can be absorbed on the carrier is limited by the surface area of the carrier [79].

The other commonly used method for cells immobilization is encapsulation which encloses cells within a thin semi-permeable membrane. The cells are free to move in the inner liquid core inside the capsule. However, the space is limited by the outer membrane [100]. In fermentation, the molecular dimensions of the microcapsules limit the growth of cells and the size of both nutrients and products. The rate of substrate transfer into the capsules will determine the rate of reaction. Encapsulation method gives several advantages such as mechanical and chemical stability of the membrane system, possibility of high loading and regulation of the fermentation reaction by selective diffusion of substrate and products [101].

There are many types of supporting materials that have been used in yeast cells immobilization such as calcium alginate, sugarcane bagasse, delignified cellulosic materials, orange peel, spent grains, corn cobs, k-carrageenan, wood blocks, porous cellulose, zeolite, loofa sponges and sorghum bagasse [102]. The support used in immobilization must be conducive to cell viability and have proper permeability for the diffusion of oxygen, essential nutrients, metabolic waste and secretory products across the polymer network. There are two types of polymers that are used as carrier in yeasts immobilization which are natural and synthetic polymers. The benefits of using natural polymers are low price and no impurities produced from chemical reaction. Synthetic polymers exhibit high chemical and biological stability, mechanical resistance to abrasion, permeable to reagents, and have large surface, capacity and porosity [103].

### 4.2 Immobilized yeasts in bioethanol production

Immobilization of yeast strains for bioethanol production is presented in Table 5. The commonly employed method for yeast immobilization is adsorption because the cells are not affected and yeast can be added or washed out from the fermentation medium [104]. Calcium alginate is the most preferred carrier due to its good biocompatibility, low cost, ease of availability [96]. Ariyajaroenwong et al., [105] reported the highest ethanol concentration of 98.48g/L by fermenting sweet sorghum juice using *S. cerevisiae* NP 01. Sorghum stalk which was used as the carrier showed an important function as the source of inoculum for ethanol production while the sweet sorghum juice which was used as the feedstocks contained essential nutrients for yeast growth. Singh et al., [106] used *S. cerevisiae* MTCC 174 to ferment sugarcane bagasse and produced only 15.4g/L of ethanol concentration. The low amount of sugar obtained from sugarcane bagasse could be the reason for low amount of ethanol concentration. Zheng et al. [107] used *S. cerevisiae* to ferment sugar molasses and obtained the highest ethanol productivity of 6.55g/L/h. The adsorption and covalent binding of MCM-41 zeolite with the embedding of alginate caused the cell in the MCM-41 mesoporous zeolite composite carrier to grow better than in pure carrier. Behera et al., [108] used *S. cerevisiae* CTCRI to ferment mahula flower and achieved ethanol productivity of only 0.27g/L/h. The lowest ethanol productivity obtained probably due to mahula flower which contains low amount of fermentable sugars compared to other types of feedstocks.

**Table 5:** Immobilization of yeasts in bioethanol production.

**Yeast strain**	**Feedstock**	**Method**	**Carrier**	**Sugar concentration (g/L)**	**Fermentation condition**	**Ethanol concentration (g/L)**	**Ethanol productivity (g/L/h)**	**References**
*S. cerevisiae*	Sugar molasses	Cross-linking and covalent binding	Alginate-based MCM-41 mesoporous zeolite composite	170.0	30 °C, 115 rpm, 12 h	78.6	6.55	[107]
*S. cerevisiae* M30	Cane molasses	Cross-linking	Bacterial cellulose-alginate (BCA) sponge	220.0	33 °C, 150 rpm, 48 h	92.0	1.92	[126]
Mutant baker's yeast 3013	Glucose and sucrose	Adsorption	Sorghum bagasse	200.0	30 °C, 16 h	92.7	5.72	[127]
*S. cerevisiae* MTCC 174	Sugarcane bagasse	Adsorption	Sugarcane bagasse	50.0	30 °C, 72 h	15.4	0.43	[106]
*S. cerevisiae* M30	Blackstrap molasses	Adsorption	Thin-shell silk cocoon	240.0	33 °C, 150 rpm, 48 h	80.6	1.85	[128]
*S. cerevisiae* DTN	Sugar beet thick juice	Adsorption	Sugar beet pulp	120.0	30 °C, 48 h	52.3	1.09	[129]
*S. cerevisiae* NP 01	Sorghum juice	Adsorption	Sweet sorghum stalks	230.0	30 °C, 72 h	98.5	1.37	[105]
*S. cerevisiae* CTCRI	Mahula flower	Entrapment	Calcium alginate	350.0	30 °C, 96 h	25.8	0.27	[108]
*S. cerevisiae var*. *ellipsoideus*	Corn meal	Entrapment	Calcium alginate	150.0	30 °C, 74 h, 150 rpm	88.9	2.34	[130]
*S. cerevisiae* T0936	Wheat straw	Entrapment	Calcium alginate	51.4	30 °C, 96 h, 150 rpm	37.1	0.38	[131]

## 5 Conclusion

Yeasts which are the most common microorganisms in bioethanol production play important function in fermenting sugars to ethanol. The influence of yeast strains, processes of fermentation and immobilization of yeasts on ethanol production has been shown in this work. Many types of yeast strains have been identified all over the world with the ability of producing ethanol from different types of feedstocks. This review paper discussed about the efficiency of different yeast strains in producing ethanol with wild-type strain as the highest ethanol producer. Fermentation process exhibited significant effect on ethanol production. Continuous SSF method has shown its ability in producing high ethanol concentration with high productivity. The application of cell immobilization in ethanol production was evaluated. Adsorption method is the preferred method of immobilizing yeast cells whereas calcium alginate is the top choice for yeasts carrier. Immobilized cells give several advantages in ethanol production such as high cell density, easy separation from the medium, high substrate conversion, less inhibition, short reaction time and cell recycling. Thus, immobilized yeasts pave better way for commercialization of bioethanol production from an economical perspective.
